# Esophagobronchial fistula - A rare complication of aluminum phosphide poisoning

**DOI:** 10.4103/1817-1737.74276

**Published:** 2011

**Authors:** Sumeet Bhargava, Rajul Rastogi, Ajay Agarwal, Gaurav Jindal

**Affiliations:** *Yash Diagnostic Center, Yash Hospital and Research Center, Civil Lines, Kanth Road, Moradabad, Uttar Pradesh, India*

**Keywords:** Aluminum phosphide, esophagobronchial fistula, poisoning

## Abstract

Aluminum phosphide is a systemic lethal poison. Fistulous communication between esophagus and airway tract (esophagorespiratory fistula) has rarely been reported in the survivors of aluminum phosphide poisoning. We report a case of benign esophagobronchial fistula secondary to aluminum phosphide poisoning, which to best of our knowledge has not been reported in the medical literature.

Aluminum phosphide is a solid fumigant used for the preservation of food grains. It is often used as a suicidal poison because of its easy availability. Aluminum phosphide poisoning, if ingested in toxic doses carries a mortality risk of approximately 80–90%. The toxic effects are due to release of highly poisonous phosphine gas.[1,2] Those who survive rarely develop fistulous communication between the esophagus and airway tract. Hence, we are reporting, probably the first case of esophagobronchial fistula secondary to aluminum phosphide poisoning.

## Case Report

A 17-year-old male presented to us with a history of acute dysphagia and severe cough following every swallow; both to liquids as well as solids. Careful history taking revealed a history of aluminum phosphide tablet ingestion 5–6 days back for which he was admitted in some other hospital, treated conservatively, and discharged uneventfully. Clinical examination of chest revealed fine crepts in lung bases while clinical examination of oral cavity and abdomen did not reveal any other significant finding. Laboratory examination, chest radiography, and ultrasonography of abdomen were unremarkable.

**Figure 1:**
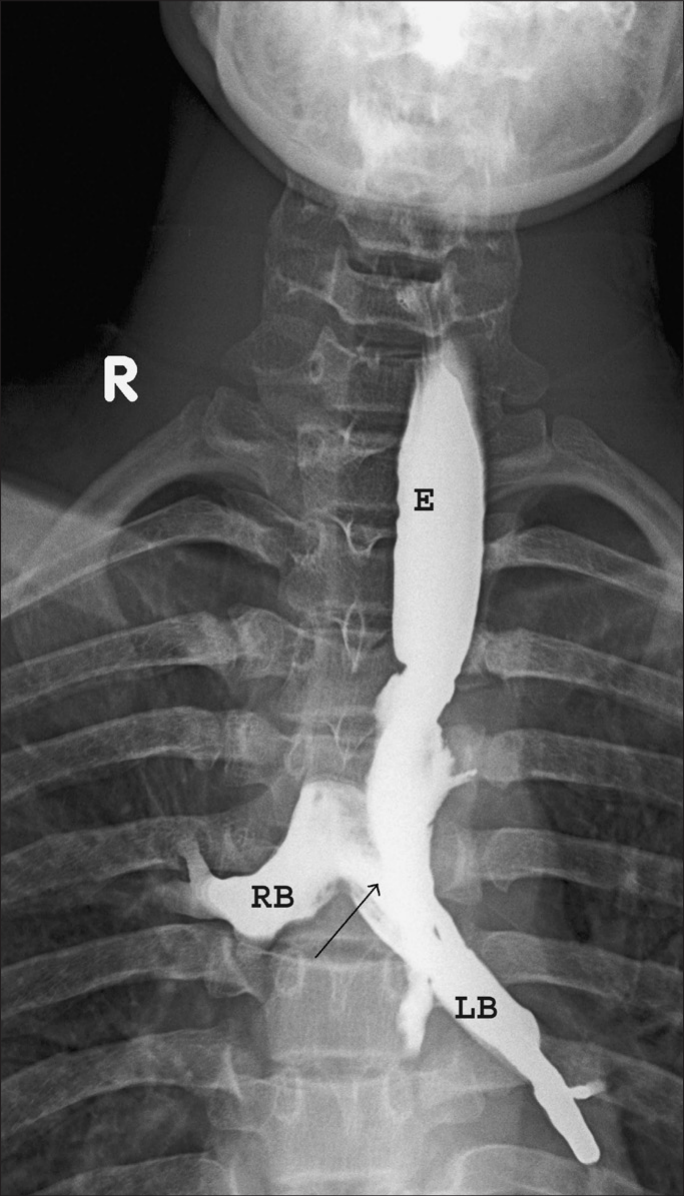
Barium esophagogram in AP projection shows narrowing of esophagus with irregular mucosa at D3 to D5 vertebral level and communication of esophagus with bronchus [black arrow]. E - Esophagus; RB -Right main bronchus, and LB - Left bronchus

Patient then underwent barium esophagogram that revealed irregular narrowing in upper esophagus (D3 to D5 vertebral level) associated with fistulous communication with left main bronchus [Figure 1]. However, due to cough the barium was seen entering the carina and right main bronchus as well. Based on the clinicoradiologic findings, the diagnosis of esophagobronchial fistula secondary to aluminum phosphide poisoning was suggested. Esophagoscopy was suggested for further work up.

Patient then underwent endoscopy at tertiary level institute that confirmed the radiological findings of a fistulous communication of esophagus with left main bronchus. The patient also underwent reconstructive surgery in the same institute with unremarkable postoperative period. Three-month postoperative follow-up of the patient at our hospital was unremarkable.

## Discussion

Aluminum phosphide is a solid fumigant, available in tablet form for use as a food-grain preservative. It releases a toxic gas, phosphine, on exposure to moisture. It is often used as an oral suicidal poison due to its easy availability. On ingestion, it produces serious systemic effects within an hour. The toxic effects and prognosis is highly dependent on dose, freshness of tablets, time-elapsed following consumption, and promptness of resuscitative procedures.

Phosphine acts by inhibiting cytochrome oxidase, a mitochondrial enzyme which is a part of a respiratory chain cycle. Acute cardiovascular collapse is the commonest mode of presentation followed by gastrointestinal, neurological, respiratory, and musculoskeletal manifestations.[1] Disseminated intravascular coagulation and renal failure are other manifestations. Local effects include severe inflammation, corrosion, and fibrosis.

There is no specific antidote of aluminum phosphide poisoning and hence the treatment is directed to reverse the signs of clinical deterioration especially cardiogenic shock, breathing difficulty, and CNS manifestations. Besides the usual resuscitative measures, intravenous magnesium sulfate has been found useful in reducing the mortality of patients, probably due to its membrane stabilizing and anti-peroxide effect.[1] Intra-aortic balloon pump (IABP) has been found to be useful in patients of aluminum phosphide poisoning presenting with cardiogenic shock.[2]

Benign strictures and tracheoesophageal fistula has been rarely reported in the medical literature in the survivors of aluminum phosphide poisoning.[3–6] Esophagobronchial fistula following aluminum phosphide poisoning as seen in our case has not been reported in the medical literature. The postulated mechanism is severe inflammation and corrosion of esophageal and tracheobronchial walls due to local release of phosphine gas secondary to local trapping or impaction of tablet in the esophageal mucosa. The potential life-threatening complication of such fistula is secondary pulmonary infection.

The sequence of swallow and cough is a classical clinical presentation in a case of fistulous communication between esophagus and airway tract, also known as *‘Ono’s sign’*. Barium esophagogram is diagnostic in majority of the cases. Esophagoscopy is needed for confirmation in few cases.

Direct repair of the fistulous openings with interposition of muscle flap is the procedure of choice. Recurrences are uncommon in benign esophagorespiratory fistula. If the esophagus is damaged beyond repair, esophageal bypass with transverse colon or stomach through retrosternal approach is the procedure of choice.[6]

To summarize, though fistulous communication between esophagus and airway tract (esophagobronchial / esophagotracheal fistula) is a rare complication seen in survivors of aluminum phosphide poisoning yet it should be kept in mind especially in those patients presenting with dysphagia. If unrecognized, it can lead to severe morbidity and even death due to aspiration and secondary pulmonary infections. Also, it is worth following survivors of aluminum phosphide poisoning patients with barium esophagogram for early detection of fistulous complication.

## References

[1] Chugh SN, Shah SN (2003). Aluminium phosphide poisoning. API Textbook of Medicine.

[2] Siddhaiah LM, Adhyapak SM, Jaydev SM, Shetty GG, Varghese K, Patil CB (2009). Intra-aortic balloon pump in toxic myocarditis due to aluminum phosphide poisoning. J Med Toxicol.

[3] Darbari A, Tandon S, Chaudhary S, Bharadwaj M, Kumar A, Singh GP (2008). Esophageal injuries due to aluminum phosphide tablet poisoning in India. Asian Cardiovasc Thorac Ann.

[4] Kapoor S, Naik S, Kumar R, Sharma S, Pruthi HS, Varshney S (2005). Benign esophageal stricture following aluminium phosphide poisoning. Indian J Gastroenterol.

[5] Tiwari J, Lahoti B, Dubey K, Mishra P, Verma S (2003). Tracheo-oesophageal fistula: an unusual complication following celphos poisoning. Indian J Surg.

[6] Darbari A, Kumar A, Chandra G, Tandon S (2007). Tracheo-oesophageal fistula with oesophageal stricture due to aluminium phosphide (celphos tablet) poisoning. Indian J Chest Dis Allied Sci.

